# 4′-*tert*-Butyl-5-chloro-3*H*-spiro­[1,3-benzothia­zole-2,1′-cyclo­hexa­ne]

**DOI:** 10.1107/S1600536812017539

**Published:** 2012-04-28

**Authors:** Mehmet Akkurt, Gökçe Cihan-Üstündağ, Gültaze Çapan, Yılmaz Dağdemir, Muhammad Nawaz Tahir

**Affiliations:** a Department of Physics, Faculty of Sciences, Erciyes University, 38039 Kayseri, Turkey; b Department of Pharmaceutical Chemistry, Faculty of Pharmacy, Istanbul University, 34116 Beyazıt, Istanbul, Turkey; c Department of Physics, University of Sargodha, Sargodha, Pakistan

## Abstract

In the title compound, C_16_H_22_ClNS, the nine-membered 2,3-dihydro-1,3-benzothia­zole ring system is essentially planar, with a maximum deviation of 0.025 (2) Å for the N atom. Its plane is almost perpendicular to the main plane of the substituted cyclo­hexane ring, which adopts a chair conformation. In the crystal, the molecules are linked by C—H⋯π inter­actions.

## Related literature

For the pharmacological activity of benzothia­zole derivatives, see: Coudert *et al.* (1988 ▶); Karalı *et al.* (2010 ▶); Palmer *et al.* (1971 ▶). For the crystal structures of similar compounds, see, for example: Akkurt *et al.* (2010 ▶); Aryai *et al.* (1976 ▶); Karalı *et al.* (2010 ▶). For standard values of bond lengths, see: Allen *et al.* (1987 ▶). For details of ring-puckering analysis, see: Cremer & Pople (1975 ▶).
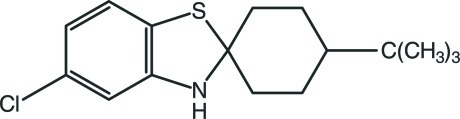


## Experimental

### 

#### Crystal data

C_16_H_22_ClNS
*M* *_r_* = 295.87
Monoclinic, 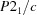

*a* = 15.2810 (18) Å
*b* = 8.9830 (8) Å
*c* = 11.8750 (13) Å
β = 109.580 (3)°
*V* = 1535.8 (3) Å^3^
*Z* = 4
Mo *K*α radiation
μ = 0.37 mm^−1^
*T* = 296 K
0.27 × 0.20 × 0.18 mm

#### Data collection

Bruker Kappa APEXII CCD diffractometer
Absorption correction: multi-scan (*SADABS*; Bruker, 2005 ▶) *T*_min_ = 0.915, *T*_max_ = 0.935
14074 measured reflections
3849 independent reflections
2330 reflections with *I* > 2σ(*I*)
*R*_int_ = 0.043

#### Refinement

*R*[*F* ^2^ > 2σ(*F* ^2^)] = 0.052
*wR*(*F* ^2^) = 0.128
*S* = 1.02
3849 reflections
179 parameters
1 restraint
H atoms treated by a mixture of independent and constrained refinement
Δρ_max_ = 0.25 e Å^−3^
Δρ_min_ = −0.24 e Å^−3^

### 

Data collection: *APEX2* (Bruker, 2009 ▶); cell refinement: *SAINT* (Bruker, 2009 ▶); data reduction: *SAINT*; program(s) used to solve structure: *SHELXS97* (Sheldrick, 2008 ▶); program(s) used to refine structure: *SHELXL97* (Sheldrick, 2008 ▶); molecular graphics: *ORTEP-3 for Windows* (Farrugia, 1997 ▶) and *PLATON* (Spek, 2009 ▶); software used to prepare material for publication: *WinGX* (Farrugia, 1999 ▶) and *PLATON*.

## Supplementary Material

Crystal structure: contains datablock(s) global, I. DOI: 10.1107/S1600536812017539/su2411sup1.cif

Structure factors: contains datablock(s) I. DOI: 10.1107/S1600536812017539/su2411Isup2.hkl

Supplementary material file. DOI: 10.1107/S1600536812017539/su2411Isup3.cml

Additional supplementary materials:  crystallographic information; 3D view; checkCIF report

## Figures and Tables

**Table 1:** Hydrogen-bond geometry (Å, °) *Cg*1 is the centroid of the C1–C6 benzene ring.

*D*—H⋯*A*	*D*—H	H⋯*A*	*D*⋯*A*	*D*—H⋯*A*
C8—H8*B*⋯*Cg*1^i^	0.97	2.84	3.796 (2)	169

Symmetry code: (i) 
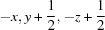
.

**Fig. 1.**
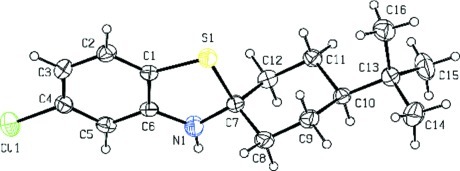
The molecular structure of the title molecule, with the atom numbering. Displacement ellipsoids are drawn at the 30% probability level.

**Fig. 2.**
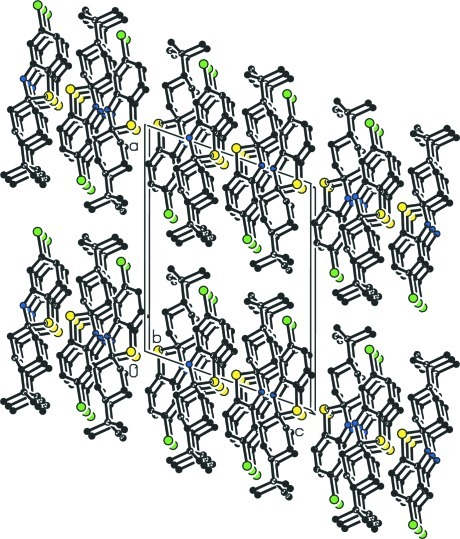
The crystal packing of the title compound viewing along b axis [H atoms have been omitted for clarity].

## supplementary crystallographic information

### Comment

The condensation of aldehydes and ketones with 2-aminothiophenoles lead to benzothiazolines and spirobenzothiazolines which are reported to exhibit antitubercular (Palmer *et al.*, 1971), analgesic (Coudert *et al.*, 1988) and antioxidant (Karalı *et al.*, 2010) properties. The reactivity of cyclic ketones towards 2-aminothiophenoles has also been examined and the structure of the end products has been discussed (Aryai *et al.*, 1976; Coudert *et al.*, 1988; Akkurt *et al.*, 2010; Karalı *et al.*, 2010). Prompted by the above observations, we report here the synthesis, spectroscopic and crystal structure of the title compound.

As shown in Fig. 1, the C7—C12 cyclohexane ring of the title compound adopts a chair conformation [puckering parameters (Cremer & Pople, 1975): Q_T_ = 0.564 (2) Å, θ = 176.5 (2) ° and φ = 4(4) °]. The mean plane of the 2,3-dihydro-1,3-benzothiazole ring system [max. deviation: -0.025 (2) Å for N1] is almost perpendicular with a dihedral angle of 89.39 (5) ° to the main plane formed by the C8,C9, C11 and C12 atoms of the cyclohexane ring. The bond lengths (Allen *et al.*, 1987) and bond angles are within the expected values.

The crystal packing is stabilized by C—H···π interactions (Table 1 and Fig. 2).

### Experimental

A mixture of 2-amino-4-chlorothiophenol (0.01 mol) and 4-*tert*-butylcyclohexanone (0.01 mol) in absolute ethanol (50 ml) was refluxed on a water bath for 8 h. The solvent was evaporated in a crystallizing dish at room temperature and the residue was recrystallized twice from ethanol, giving X-ray quality crystals [Yield: 24.3%, m.p.: 453–455 K]. Analysis calculated for C_16_H_22_ClNS: C 64.95, H 7.49, N 4.73%. Found: C 64.91, H 7.47, N 4.64%. Spectroscopic data for the title compound are given in the archive CIF.

### Refinement

The NH H atom was located in a difference Fourier map and freely refined. C-bound H atoms were placed in calculated positions and treated as riding atoms : C—H = 0.93, 0.96, 0.97 and 0.98 Å, for the aromatic, methyl, methylene and methine H atoms, respectively, with *U*_iso_(H) = *xU*_eq_(C), *x* = 1.5 for methyl H atoms and = 1.2 for other H atoms.

### Crystal data

**Table:**

C_16_H_22_ClNS	*F*(000) = 632
*M**_r_* = 295.87	*D*_x_ = 1.280 Mg m^−^^3^
Monoclinic, *P*2_1_/*c*	Mo *K*α radiation, λ = 0.71073 Å
Hall symbol: -P 2ybc	Cell parameters from 776 reflections
*a* = 15.2810 (18) Å	θ = 3.3–19.5°
*b* = 8.9830 (8) Å	µ = 0.37 mm^−^^1^
*c* = 11.8750 (13) Å	*T* = 296 K
β = 109.580 (3)°	Prism, colourless
*V* = 1535.8 (3) Å^3^	0.27 × 0.20 × 0.18 mm
*Z* = 4	

### Data collection

**Table:**

Bruker Kappa APEXII CCD diffractometer	3849 independent reflections
Radiation source: fine-focus sealed tube	2330 reflections with *I* > 2σ(*I*)
Graphite monochromator	*R*_int_ = 0.043
ω scans	θ_max_ = 28.5°, θ_min_ = 2.7°
Absorption correction: multi-scan (*SADABS*; Bruker, 2005)	*h* = −20→20
*T*_min_ = 0.915, *T*_max_ = 0.935	*k* = −11→10
14074 measured reflections	*l* = −15→15

### Refinement

**Table:**

Refinement on *F*^2^	Primary atom site location: structure-invariant direct methods
Least-squares matrix: full	Secondary atom site location: difference Fourier map
*R*[*F*^2^ > 2σ(*F*^2^)] = 0.052	Hydrogen site location: inferred from neighbouring sites
*wR*(*F*^2^) = 0.128	H atoms treated by a mixture of independent and constrained refinement
*S* = 1.02	*w* = 1/[σ^2^(*F*_o_^2^) + (0.0473*P*)^2^ + 0.2285*P*] where *P* = (*F*_o_^2^ + 2*F*_c_^2^)/3
3849 reflections	(Δ/σ)_max_ < 0.001
179 parameters	Δρ_max_ = 0.25 e Å^−^^3^
1 restraint	Δρ_min_ = −0.24 e Å^−^^3^

### Special details

**Table:**

Experimental. Spectroscopic data for the title compound: IR (KBr) ν = 3370 (N—H), 2962, 2912, 2862 (C—H), 1585, 1571, 1473, 1442 (C=C) cm^-1^; 1*H*-NMR (DMSO-d6, 500 MHz) d= 0.83–0.86 (9*H*, m, 4'-C(CH3)3-cyc.), 0.95–1.02 (1*H*, m, CH/CH2-cyc.), 1.09–1.36 (2*H*, m, CH/CH2-cyc.), 1.58–1.72 (4*H*, m, CH/CH2-cyc.), 2.15–2.22 (2*H*, m, CH/CH2-cyc.), 6.40, 6.47 (1*H*, 2 d, J=2.0 Hz, H4-bt.), 6.50 (1*H*, dd, J=8.1, 2.0 Hz, H6-bt.), 6.90 (1*H*, d, J=7.8 Hz, H7-bt.), 6.73, 6.97 (1*H*, 2 s, NH) p.p.m. (cyc.=cyclohexane, bt.=benzothiazole).
Geometry. Bond distances, angles *etc*. have been calculated using the rounded fractional coordinates. All su's are estimated from the variances of the (full) variance-covariance matrix. The cell e.s.d.'s are taken into account in the estimation of distances, angles and torsion angles
Refinement. Refinement on *F*^2^ for ALL reflections except those flagged by the user for potential systematic errors. Weighted *R*-factors *wR* and all goodnesses of fit *S* are based on *F*^2^, conventional *R*-factors *R* are based on *F*, with *F* set to zero for negative *F*^2^. The observed criterion of *F*^2^ > σ(*F*^2^) is used only for calculating -*R*-factor-obs *etc*. and is not relevant to the choice of reflections for refinement. *R*-factors based on *F*^2^ are statistically about twice as large as those based on *F*, and *R*-factors based on ALL data will be even larger.

### Fractional atomic coordinates and isotropic or equivalent isotropic displacement parameters (Å^2^)

**Table:**

	*x*	*y*	*z*	*U*_iso_*/*U*_eq_	
Cl1	−0.35486 (5)	0.80459 (8)	0.13722 (7)	0.0761 (3)	
S1	0.02827 (4)	0.69814 (6)	0.07426 (5)	0.0503 (2)	
N1	−0.00626 (14)	0.8895 (2)	0.2213 (2)	0.0628 (8)	
C1	−0.08493 (14)	0.7159 (2)	0.07911 (17)	0.0369 (7)	
C2	−0.16334 (16)	0.6409 (2)	0.0125 (2)	0.0474 (8)	
C3	−0.24750 (16)	0.6684 (2)	0.0286 (2)	0.0515 (8)	
C4	−0.25028 (16)	0.7710 (2)	0.1132 (2)	0.0469 (8)	
C5	−0.17272 (16)	0.8482 (2)	0.1807 (2)	0.0447 (7)	
C6	−0.08920 (15)	0.8210 (2)	0.16337 (18)	0.0390 (7)	
C7	0.07535 (15)	0.8422 (2)	0.1936 (2)	0.0442 (7)	
C8	0.11774 (16)	0.9705 (2)	0.1463 (2)	0.0519 (8)	
C9	0.20566 (15)	0.9285 (2)	0.1218 (2)	0.0500 (8)	
C10	0.27931 (14)	0.8647 (2)	0.23261 (18)	0.0408 (7)	
C11	0.23620 (15)	0.7316 (2)	0.27578 (19)	0.0438 (7)	
C12	0.14820 (15)	0.7730 (2)	0.30136 (19)	0.0467 (8)	
C13	0.37389 (16)	0.8291 (2)	0.2176 (2)	0.0509 (8)	
C14	0.4048 (2)	0.9596 (3)	0.1572 (3)	0.0845 (14)	
C15	0.44715 (18)	0.8047 (3)	0.3408 (2)	0.0800 (11)	
C16	0.36933 (18)	0.6896 (3)	0.1415 (2)	0.0651 (10)	
H1N	−0.0005 (17)	0.955 (2)	0.2742 (16)	0.070 (8)*	
H2	−0.16000	0.57120	−0.04380	0.0570*	
H3	−0.30100	0.61850	−0.01690	0.0620*	
H5	−0.17650	0.91750	0.23700	0.0540*	
H8A	0.07240	1.00700	0.07300	0.0620*	
H8B	0.13160	1.05100	0.20410	0.0620*	
H9A	0.23040	1.01600	0.09520	0.0600*	
H9B	0.19090	0.85550	0.05810	0.0600*	
H10	0.29130	0.94100	0.29500	0.0490*	
H11A	0.22180	0.65430	0.21530	0.0520*	
H11B	0.28120	0.69160	0.34790	0.0520*	
H12A	0.12260	0.68450	0.32520	0.0560*	
H12B	0.16360	0.84290	0.36740	0.0560*	
H14A	0.46750	0.94360	0.15950	0.1270*	
H14B	0.40160	1.05020	0.19850	0.1270*	
H14C	0.36470	0.96690	0.07550	0.1270*	
H15A	0.50660	0.78790	0.33200	0.1200*	
H15B	0.43040	0.71970	0.37820	0.1200*	
H15C	0.45040	0.89120	0.38960	0.1200*	
H16A	0.31930	0.69930	0.06700	0.0980*	
H16B	0.35900	0.60370	0.18350	0.0980*	
H16C	0.42680	0.67840	0.12620	0.0980*	

### Atomic displacement parameters (Å^2^)

**Table:**

	*U*^11^	*U*^22^	*U*^33^	*U*^12^	*U*^13^	*U*^23^
Cl1	0.0543 (4)	0.0816 (5)	0.1046 (6)	0.0027 (3)	0.0428 (4)	−0.0018 (4)
S1	0.0456 (3)	0.0510 (4)	0.0568 (4)	−0.0017 (3)	0.0203 (3)	−0.0182 (3)
N1	0.0467 (12)	0.0636 (14)	0.0770 (15)	−0.0030 (10)	0.0191 (11)	−0.0390 (12)
C1	0.0441 (12)	0.0319 (11)	0.0363 (11)	0.0025 (9)	0.0158 (9)	0.0017 (8)
C2	0.0533 (14)	0.0404 (12)	0.0483 (13)	−0.0035 (10)	0.0168 (11)	−0.0086 (10)
C3	0.0460 (14)	0.0488 (14)	0.0571 (15)	−0.0096 (11)	0.0139 (12)	−0.0054 (11)
C4	0.0438 (13)	0.0414 (12)	0.0586 (15)	0.0046 (10)	0.0214 (11)	0.0072 (10)
C5	0.0531 (14)	0.0361 (11)	0.0492 (13)	0.0061 (10)	0.0229 (11)	−0.0011 (9)
C6	0.0455 (13)	0.0295 (10)	0.0412 (12)	0.0022 (9)	0.0133 (10)	−0.0016 (9)
C7	0.0402 (13)	0.0395 (12)	0.0517 (13)	−0.0018 (9)	0.0139 (11)	−0.0117 (10)
C8	0.0483 (14)	0.0382 (12)	0.0580 (15)	0.0040 (10)	0.0030 (11)	0.0069 (10)
C9	0.0518 (14)	0.0434 (12)	0.0515 (14)	−0.0031 (10)	0.0131 (11)	0.0118 (10)
C10	0.0424 (12)	0.0352 (11)	0.0411 (12)	−0.0016 (9)	0.0091 (10)	−0.0016 (9)
C11	0.0465 (13)	0.0404 (12)	0.0400 (12)	0.0039 (10)	0.0087 (10)	0.0094 (9)
C12	0.0554 (15)	0.0414 (12)	0.0440 (13)	−0.0032 (10)	0.0176 (11)	−0.0010 (9)
C13	0.0451 (14)	0.0488 (13)	0.0572 (15)	0.0010 (10)	0.0151 (11)	−0.0043 (11)
C14	0.070 (2)	0.0732 (19)	0.126 (3)	−0.0153 (15)	0.0536 (19)	0.0001 (18)
C15	0.0468 (16)	0.105 (2)	0.077 (2)	0.0111 (15)	0.0061 (15)	−0.0186 (16)
C16	0.0638 (17)	0.0669 (16)	0.0687 (17)	0.0053 (13)	0.0278 (14)	−0.0127 (13)

### Geometric parameters (Å, º)

**Table:**

Cl1—C4	1.741 (3)	C2—H2	0.9300
S1—C1	1.757 (2)	C3—H3	0.9300
S1—C7	1.875 (2)	C5—H5	0.9300
N1—C6	1.369 (3)	C8—H8A	0.9700
N1—C7	1.456 (3)	C8—H8B	0.9700
N1—H1N	0.844 (18)	C9—H9A	0.9700
C1—C2	1.371 (3)	C9—H9B	0.9700
C1—C6	1.393 (3)	C10—H10	0.9800
C2—C3	1.384 (4)	C11—H11A	0.9700
C3—C4	1.375 (3)	C11—H11B	0.9700
C4—C5	1.375 (3)	C12—H12A	0.9700
C5—C6	1.381 (3)	C12—H12B	0.9700
C7—C12	1.519 (3)	C14—H14A	0.9600
C7—C8	1.519 (3)	C14—H14B	0.9600
C8—C9	1.515 (3)	C14—H14C	0.9600
C9—C10	1.528 (3)	C15—H15A	0.9600
C10—C13	1.548 (3)	C15—H15B	0.9600
C10—C11	1.534 (3)	C15—H15C	0.9600
C11—C12	1.521 (3)	C16—H16A	0.9600
C13—C15	1.531 (3)	C16—H16B	0.9600
C13—C16	1.533 (3)	C16—H16C	0.9600
C13—C14	1.529 (4)		
			
C1—S1—C7	92.59 (10)	C7—C8—H8A	109.00
C6—N1—C7	118.31 (19)	C7—C8—H8B	109.00
C6—N1—H1N	122.4 (18)	C9—C8—H8A	109.00
C7—N1—H1N	119.2 (18)	C9—C8—H8B	109.00
C2—C1—C6	120.3 (2)	H8A—C8—H8B	108.00
S1—C1—C6	111.63 (16)	C8—C9—H9A	109.00
S1—C1—C2	128.03 (16)	C8—C9—H9B	109.00
C1—C2—C3	120.35 (19)	C10—C9—H9A	109.00
C2—C3—C4	118.6 (2)	C10—C9—H9B	109.00
Cl1—C4—C3	119.39 (19)	H9A—C9—H9B	108.00
Cl1—C4—C5	118.43 (17)	C9—C10—H10	107.00
C3—C4—C5	122.2 (2)	C11—C10—H10	107.00
C4—C5—C6	118.9 (2)	C13—C10—H10	107.00
C1—C6—C5	119.7 (2)	C10—C11—H11A	109.00
N1—C6—C1	114.0 (2)	C10—C11—H11B	109.00
N1—C6—C5	126.29 (19)	C12—C11—H11A	109.00
S1—C7—N1	103.38 (15)	C12—C11—H11B	109.00
N1—C7—C8	111.46 (17)	H11A—C11—H11B	108.00
S1—C7—C8	110.32 (15)	C7—C12—H12A	109.00
S1—C7—C12	109.96 (13)	C7—C12—H12B	109.00
C8—C7—C12	109.82 (19)	C11—C12—H12A	109.00
N1—C7—C12	111.75 (19)	C11—C12—H12B	109.00
C7—C8—C9	113.43 (16)	H12A—C12—H12B	108.00
C8—C9—C10	111.89 (18)	C13—C14—H14A	110.00
C9—C10—C11	107.78 (18)	C13—C14—H14B	109.00
C9—C10—C13	115.16 (18)	C13—C14—H14C	109.00
C11—C10—C13	113.56 (16)	H14A—C14—H14B	109.00
C10—C11—C12	112.56 (16)	H14A—C14—H14C	109.00
C7—C12—C11	112.28 (18)	H14B—C14—H14C	109.00
C10—C13—C15	109.36 (19)	C13—C15—H15A	110.00
C10—C13—C16	112.15 (19)	C13—C15—H15B	109.00
C14—C13—C16	108.1 (2)	C13—C15—H15C	110.00
C15—C13—C16	108.70 (18)	H15A—C15—H15B	110.00
C14—C13—C15	108.4 (2)	H15A—C15—H15C	109.00
C10—C13—C14	110.04 (18)	H15B—C15—H15C	109.00
C1—C2—H2	120.00	C13—C16—H16A	110.00
C3—C2—H2	120.00	C13—C16—H16B	109.00
C2—C3—H3	121.00	C13—C16—H16C	109.00
C4—C3—H3	121.00	H16A—C16—H16B	109.00
C4—C5—H5	121.00	H16A—C16—H16C	109.00
C6—C5—H5	121.00	H16B—C16—H16C	110.00
			
C7—S1—C1—C2	180.00 (19)	C4—C5—C6—N1	178.9 (2)
C7—S1—C1—C6	−0.14 (15)	C4—C5—C6—C1	−0.5 (3)
C1—S1—C7—N1	−1.11 (14)	S1—C7—C8—C9	−68.5 (2)
C1—S1—C7—C8	−120.40 (16)	N1—C7—C8—C9	177.21 (19)
C1—S1—C7—C12	118.33 (16)	C12—C7—C8—C9	52.8 (2)
C6—N1—C7—S1	2.3 (2)	S1—C7—C12—C11	69.25 (19)
C6—N1—C7—C8	120.8 (2)	N1—C7—C12—C11	−176.55 (16)
C6—N1—C7—C12	−115.9 (2)	C8—C7—C12—C11	−52.3 (2)
C7—N1—C6—C1	−2.6 (3)	C7—C8—C9—C10	−56.6 (2)
C7—N1—C6—C5	178.01 (19)	C8—C9—C10—C11	56.2 (2)
S1—C1—C2—C3	179.60 (16)	C8—C9—C10—C13	−175.93 (15)
C6—C1—C2—C3	−0.3 (3)	C9—C10—C11—C12	−56.7 (2)
S1—C1—C6—N1	1.5 (2)	C13—C10—C11—C12	174.45 (17)
S1—C1—C6—C5	−179.09 (15)	C9—C10—C13—C14	46.7 (2)
C2—C1—C6—N1	−178.61 (18)	C9—C10—C13—C15	165.71 (17)
C2—C1—C6—C5	0.8 (3)	C9—C10—C13—C16	−73.6 (2)
C1—C2—C3—C4	−0.6 (3)	C11—C10—C13—C14	171.7 (2)
C2—C3—C4—C5	0.9 (3)	C11—C10—C13—C15	−69.4 (2)
C2—C3—C4—Cl1	−178.66 (16)	C11—C10—C13—C16	51.3 (2)
Cl1—C4—C5—C6	179.20 (15)	C10—C11—C12—C7	56.6 (2)
C3—C4—C5—C6	−0.4 (3)		

### Hydrogen-bond geometry (Å, º)

Cg1 is the centroid of the C1–C6 benzene ring.

**Table:**

*D*—H···*A*	*D*—H	H···*A*	*D*···*A*	*D*—H···*A*
C8—H8*B*···*Cg*1^i^	0.97	2.84	3.796 (2)	169

Symmetry code: (i) −*x*, *y*+1/2, −*z*+1/2.
